# SwissScoring: a nationwide survey about SAPS II assessing accuracy

**DOI:** 10.1186/cc13250

**Published:** 2014-03-17

**Authors:** M Previsdomini, B Cerutti, P Merlani, HU Rothen, M Kaufmann, A Perren

**Affiliations:** 1 Ospedale Regionale Bellinzona e Valli, Bellinzona, Switzerland; 2 Unit of Development and Research in Medical Education, Geneva, Switzerland; 3 Ospedale Regionale, Lugano, Switzerland; 4 Bern University Hospital, Bern, Switzerland; 5 University Hospital Basel, Switzerland

## Introduction

The first description of SAPS II dates back to 1993 [1], but little is known about the scoring accuracy in daily practice and factors possibly affecting it. The purpose of this study was to evaluate accuracy of SAPS II scoring by means of a nationwide survey.

## Methods

Twenty clinical scenarios, covering a broad range of illness severity, were randomly assigned to a convenience sample of clinicians or nurses of Swiss adult ICUs. They were asked to assign a SAPS II score (including details for each item of the score) for one scenario. Results were compared with a reference, as defined by five experienced researchers using a Delphi method, and cross-matched with data related to training and quality control for scoring, information on daily practice of scoring, and structural and organizational properties of each participating ICU.

## Results

Sixty-three (81%) of 78 adult ICUs participated in this survey. A perfect match with each single reference item was found in 27 (7.8%) of 345 scorings. The participants' mean SAPS II scoring was 42.6 ± 23.4, with a bias of +5.7 (95% CI 2.0 to 9.5) as compared with the reference score. There was no evidence of variation of the bias according to case severity, number of beds in the unit, number of residents during workshifts, linguistic area, profession (physician vs. nurse), experience, initial SAPS II training, and presence of a quality control system. The items with highest scoring accuracy were bilirubin, temperature and chronic disease (93%, 93% and 91% respectively), whereas the lowest agreement was found for urinary output and for the Glasgow Coma Scale (63% and 64%).

## Conclusion

This nationwide survey suggests a wide variability of SAPS II scoring results. On average, SAPS II was overestimated by more than 10%, irrespective of the profession or experience of the scorer or the structural characteristics of the ICUs. At least one person per unit involved in the scoring should be trained by the national society and should be responsible for the scoring quality.
